# Comparison of sex differences in cognitive function in older adults between high- and middle-income countries and the role of education: a population-based multicohort study

**DOI:** 10.1093/ageing/afad019

**Published:** 2023-02-21

**Authors:** Mikaela Bloomberg, Aline Dugravot, Andrew Sommerlad, Mika Kivimäki, Archana Singh-Manoux, Séverine Sabia

**Affiliations:** Department of Epidemiology and Public Health, University College London, UK; Université Paris Cité, Inserm U1153, Epidemiology of Ageing and Neurodegenerative diseases, France; Division of Psychiatry, University College London, UK; Department of Epidemiology and Public Health, University College London, UK; Department of Epidemiology and Public Health, University College London, UK; Université Paris Cité, Inserm U1153, Epidemiology of Ageing and Neurodegenerative diseases, France; Department of Epidemiology and Public Health, University College London, UK; Université Paris Cité, Inserm U1153, Epidemiology of Ageing and Neurodegenerative diseases, France

**Keywords:** cognitive function, cognitive aging, cognition, health inequalities, comparative study, older people

## Abstract

**Background:**

The extent to which education explains variations in sex differences in cognitive function between countries at different levels of economic development is unknown. We examined the role of education in sex differences in four cognitive domains in high- and middle-income countries.

**Methods:**

Analyses were based on 70,846 participants, aged 60 years and older, in cohort studies from a high-income (United States) and four middle-income countries (Mexico, Brazil, China, and India). We used weighted linear models to allow nationally-representative comparisons of sex differences in orientation, memory, attention, and fluency using the United States as the reference, before and after adjustment for education, and after stratification by education.

**Results:**

Females had lower levels of education than males in all countries, particularly in India. Before adjustment for education, sex differences in orientation and attention in all middle-income countries, memory in Brazil, China, and India, and fluency in India were less favourable to females than in the United States (*P* < 0.010). For example, females outperformed males in memory in the United States (mean difference [male–female scores] = −0.26 standard deviations [95% CI −0.30, −0.22]) but not in China (0.15 [0.09, 0.21]) or India (0.16 [0.13, 0.19]). Adjustment for education attenuated these sex differences. In analyses stratified by education, there were minimal sex differences in the high education group in all countries.

**Conclusion:**

Education contributes to larger female disadvantages in cognitive function at older ages in middle-income countries compared with the United States. Gender equity in education is an important target to reduce sex disparities in cognitive function globally.

## Key Points

- Education is an important determinant of cognitive function; sex disparities in education differ by economic development of a country and may explain variations in sex differences in cognitive function in older adults across countries.
- We found that lower education levels among females in middle-income compared with high-income countries contribute to sex differences in cognitive function at older ages that are more unfavourable to females in middle-income countries.
- Improving equity in access to education and level of education for women may reduce the cognitive disadvantages experienced by women at older ages in middle- and high-income countries
- National and international policies on cognitive health ought to prioritise the promotion of education, with an emphasis on gender equity, to improve cognitive outcomes for all.

## Introduction

With population ageing [1], improvement in life expectancy is not consistently accompanied by commensurate improvements in health for all older adults [2]. There are substantial within-country sociodemographic disparities in health at older ages, and further differences between countries at different levels of economic development [3]. In general terms, females outlive males but often in poorer health [4], including age-related outcomes such as dementia [5]. Cognitive dysfunction is a characteristic feature of dementia and an important predictor of loss of autonomy and poor quality of life at older ages [6]. There are well-documented sex differences in cognitive function [7] that may vary as a function of the economic development of the country [8, 9], due to environmental exposures differing in lower-income compared with high-income countries.

Findings from high-income countries suggest that on average males outperform females on tests of visuospatial ability and attention, while females outperform males on episodic memory and some verbal tasks [7, 9, 10]. Sex differences in cognitive function are likely due to a combination of biological differences [11, 12], as well as social and economic factors. Females have had limited access to education historically [13], which may contribute to sex differences in cognitive function as education confers a cognitive advantage that persists throughout life [14, 15]. Education may therefore partly explain sex differences in cognitive function in high- [16, 17] and middle-income countries [8, 9, 18–20], with larger female cognitive disadvantages in lower-income countries [21], even in cognitive domains such as memory where females outperform males in high-income countries [20]. Previous studies that used data from both high- and middle-income countries to examine the role of education in sex differences in cognitive function were based on non-representative samples [8, 9] that are prone to selection bias, precluding cross-national comparison and generalisation.

Using data on persons aged 60+ from five nationally-representative cohort studies, we compared sex differences in four cognitive domains (orientation, episodic memory, attention, and verbal fluency) between a high-income country (United States) and four middle-income countries (Mexico, Brazil, China, and India), and examined the role of education in these sex differences. We hypothesised that differences between countries in educational sex inequalities would contribute to between-country differences in sex inequalities in cognitive function at older ages.

## Methods

### Data sources

Data were drawn from participants aged 60+ from the US-based Health and Retirement Study (HRS) [22], the Mexican Health and Aging Study (MHAS) [23], the Brazilian Longitudinal Study on Aging (ELSI) [24], the China Health and Retirement Longitudinal Study (CHARLS) [25], and the Longitudinal Aging Study in India (LASI) [26]. The United States served as the reference high-income country, whereas Mexico, Brazil, China, and India are middle-income countries, as defined by the World Bank [27]. The distribution of number of years of schooling by sex in the United States is generally similar to other high-income countries [28]. Middle-income countries were selected based on availability of cognitive data. These studies are designed to facilitate cross-national comparisons; details of survey design are described elsewhere [22–26]. To minimise period effects, one wave of each study was used from the 5-year period from 2015-2019: HRS wave 13 (2016-18), MHAS wave 5 (2018-19), ELSI wave 1 (2015), CHARLS wave 4 (2018), and LASI wave 1 (2017-19). In each study, cross-sectional weights are supplied for each respondent to allow nationally representative estimates, designed to account for multistage sampling strategies and non-response.

### Sex and covariates

Sex was reported by participants as male or female. Other sociodemographic covariates included age, marital status (married/cohabiting or not), and country (United States [HRS] as the reference category, Mexico [MHAS], Brazil [ELSI], China [CHARLS], and India [LASI]).

Education in HRS was categorised into low (below high school diploma), intermediate (high school diploma or equivalent), and high (above high school diploma). In the other cohorts, participants were grouped into education categories such that the distribution of low, intermediate, and high education in each cohort was approximately similar to the distribution in HRS (Appendix 1).

### Cognitive function

Four cognitive domains were examined: orientation (date naming); episodic memory, (immediate/delayed recall); attention (serial 7s); and verbal fluency (animal naming); see Appendix 2 for details. Orientation and episodic memory were available in all cohorts. Attention was not tested in ELSI. Verbal fluency was not tested in CHARLS.

### Statistical analysis

Missing data were imputed using multiple imputation with chained equations (50 imputations). Details of the imputation method and patterns of missingness are described in Appendices 3–4. Cognitive scores were standardised in each cohort to allow comparison.

Sex differences in education in each country were examined using a weighted ordinal logistic model with education level as the outcome. To examine whether sex differences in education in middle-income countries were larger than in the United States, we pooled data from all cohort studies and included sex, age, country, and interaction between sex and country as predictors, with HRS as the reference.

We used weighted linear regression to examine sex differences in each cognitive domain. The interaction between sex and age terms (age, age^2^) suggested no robust change in sex differences with age (*P* > 0.05, apart for memory in India), allowing us to exclude these interaction terms and conduct analyses on the entire study population.

Sex differences in the four cognitive domains in each country were examined by first pooling data from all cohorts. These models included sex, age (centred at 65 years), age^2^, marital status, country, and interactions of country with sex, age, age^2^, and marital status. These analyses were then further adjusted for education, interactions between education and age terms, and education and country. Sex differences in each of the middle-income countries were compared with the United States, with *P*-values for interactions between sex and country reported in the results.

**Table 1:** Characteristics of males and females in five countries after imputation of missing data and weighting to obtain national representativeness

	United States (HRS)			Mexico (MHAS)			Brazil (ELSI)			China (CHARLS)			India (LASI)		
	Males	Females	*P*-value	Males	Females	*P*-value	Males	Females	*P*-value	Males	Females	*P*-value	Males	Females	*P*-value
	45.4%	54.6%		45.8%	54.2%		44.2%	55.8%		49.3%	50.7%		49.2%	50.8%	
Age, mean	70.4	71.7	<0.001	70.5	70.1	0.23	69.4	70.3	0.01	69.7	70.1	0.05	68.7	68.7	0.71
Age group															
60–69	54.0	50.1		53.1	56.4		59.0	54.7		57.4	56.8		62.0	61.5	
70–79	30.4	29.4	<0.001	31.0	28.9	0.25	29.3	30.3	0.004	30.2	28.7	0.08	28.2	27.5	0.01
80+	15.6	20.5		15.9	14.7		11.7	15.0		12.4	14.5		9.8	11.0	
Married/partnered															
Yes	74.1	53.0	<0.001	79.3	50.8	<0.001	75.0	44.3	<0.001	85.7	67.8	<0.001	82.4	46.0	<0.001
No	25.9	47.0		20.7	49.2		25.0	55.7		14.3	32.2		17.6	54.0	
Education															
Low	12.5	14.1		18.7	24.9		25.7	29.0		14.0	45.6		44.4	75.7	
Intermediate	47.5	53.7	<0.001	52.8	49.8	<0.001	53.0	50.7	0.08	48.9	35.6	<0.001	25.5	15.3	<0.001
High	40.0	32.2		28.5	25.3		21.3	20.3		37.1	18.8		30.1	9.0	

Data shown are percentages unless otherwise indicated.

Abbreviations: HRS, Health and Retirement Study; MHAS, Mexican Health and Aging Study; ELSI, Brazilian Longitudinal Study of Aging; CHARLS, Chinese Health and Retirement Longitudinal Study; LASI, Longitudinal Aging Study in India

We then examined whether sex differences varied by education group in each country. Analyses were undertaken separately in each country and included sex, age, age^2^, marital status, education, interactions between education and age terms, and between education and sex. Then, analyses were stratified by education in order to report sex differences in each education group.

**Table 2:** Odds ratio of being in higher education group for females compared with males

Country (cohort)	Odds ratio (95% CI)^a^	*P*-value^b^
United States (HRS)	0.83 (0.77, 0.90)	Ref.
Mexico (MHAS)	0.77 (0.66, 0.89)	0.36
Brazil (ELSI)	0.94 (0.83, 1.05)	0.10
China (CHARLS)	0.26 (0.23, 0.29)	<0.001
India (LASI)	0.17 (0.16, 0.19)	<0.001

^a^Odds ratio below 1 indicates females are less likely to be in higher education group compared with males, estimated using weighted ordinal logistic models with education level (1 = low, 2 = intermediate, 3 = high) as the outcome.

^b^ *P*-value<0.05 indicates that sex difference in the education level for the given country differs from that in the United States.

Abbreviations: HRS, Health and Retirement Study; MHAS, Mexican Health and Aging Study; ELSI, Brazilian Longitudinal Study of Aging; CHARLS, Chinese Health and Retirement Longitudinal Study; LASI, Longitudinal Aging Study in India

Finally, we examined whether sex differences in cognitive function in each education group differed between countries using the pooled dataset. Analyses were stratified by education and included sex, age, age^2^, marital status, country, and interactions between country and each of the other covariates. For each education category, we reported the *P*-values for the interactions between sex and country to examine whether sex differences in the middle-income countries differed from the United States. Analyses were undertaken using Stata 17 with a two-sided *P* < 0.05 considered statistically significant.

## Results

Analyses were based on 70,846 participants aged 60+, including 13,590 from the United States, 10,121 from Mexico, 5,432 from Brazil, 10,226 from China, and 31,477 from India. Characteristics of participants in each country based on weighted, imputed data are shown in Table 1, with corresponding observed data in Appendix 5. There were negligible sex differences (<1.5 years) in mean age across the five countries. Females were less likely than males to be married/cohabiting and less likely to have received a high level of education in all countries (*P* < 0.001) except Brazil (*P* = 0.08). The latter disadvantage was larger in China and India (*P* < 0.001) than the United States (Table 2).

**Figure 1:**
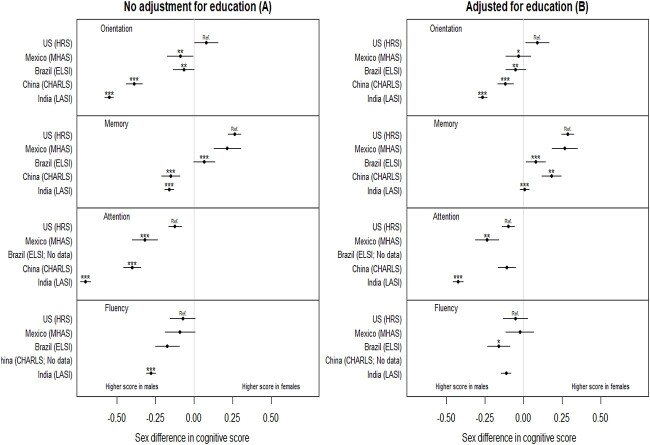
Sex differences in standardised cognitive scores in each country. The left panel (A) shows sex differences in standardised cognitive scores in each country. The right panel (B) shows analyses further adjusted for education. The confidence interval for each estimate indicates whether sex differences are statistically significant. Tests for difference with the United States (HRS) were based on pooled data; asterisks were used to indicate whether the sex difference in a given country differs from the sex difference in the United States as follows: ^*^statistically significant at α = 0.05, ^*^^*^α = 0.01, ^*^^*^^*^α = 0.001.

Sex differences in cognitive scores in each country before and after adjustment for education are shown in Figure 1(A) and (B), respectively; the figures also show *P*-values of comparison of sex differences between countries using the United States as the reference. Before adjustment for education, females had higher scores on orientation than males (female–male standardised score [95% confidence interval] = 0.08 [0.15, 0.00]) in the United States, whereas males outperformed females in Mexico (−0.09 [−0.17, 0.00]), Brazil (−0.07 [−0.13, 0.00]), China (−0.39 [−0.44, −0.33]), and India (−0.55 [−0.58, −0.52]). After adjustment for education, the female disadvantage persisted only in China (−0.12 [−0.17, −0.07]) and India (−0.27 [−0.29, −0.24]). Pooled data showed sex differences in middle-income countries differed from those in the United States both before (*P* < 0.01 for all) and after adjustment for education (*P* < 0.05 for all).

Before adjustment for education, sex differences in memory in Brazil, China, and India differed from the United States (*P* < 0.001 for all). Females performed better than males in the United States (0.26 [0.22, 0.30]), Mexico (0.21 [0.13, 0.30]), and Brazil (0.07 [0.00, 0.13]), but worse in China (−0.15 [−0.21, −0.09]) and India (−0.16 [−0.19, −0.13]). After adjustment for education, there was no sex difference in memory in India and a female advantage in all other countries, although the female advantage in the United States remained larger than in Brazil (*P* < 0.001), China (*P* < 0.01), and India (*P* < 0.001).

For attention, males outperformed females before adjustment for education in all countries, but the magnitude of the female disadvantage was larger in the middle-income countries (−0.32 [−0.40, −0.24] in Mexico, −0.40 [−0.46, −0.34] in China, and − 0.70 [−0.74, −0.67] in India), than in the United States (−0.12 [−0.17, −0.08]; *P* < 0.001 for all comparisons). After adjustment for education, the female disadvantage in attention was attenuated in all countries, but remained larger in Mexico (−0.24 [−0.31, −0.16]) and India (−0.43 [−0.46, −0.39]) than in the United States (−0.10 [−0.14, −0.06]; *P* for comparison<0.01 for Mexico; *P* < 0.001 for India).

For fluency, there was a female disadvantage before adjustment for education in the United States (−0.07 [−0.15, 0.01]) and Mexico (−0.09 [−0.19, 0.01]), though these sex differences did not reach statistical significance. A female disadvantage was found in Brazil (−0.17 [−0.25, −0.10]) and India (−0.28 [−0.31, −0.25]), with the sex difference in India differing from that in the United States (*P* < 0.001). After adjustment for education, the female disadvantage in Brazil (−0.16 [−0.24, −0.09]) was larger than that in the United States (*P* < 0.05), whereas the female disadvantage in India (−0.11 [−0.14, −0.08]) was attenuated such that it no longer differed from the United States (−0.05 [−0.13, 0.03]).

In the United States and Mexico, sex differences in all cognitive scores were similar in the three education groups, although a qualitative trend towards smaller differences in the high education category was observed (Table 3). The interaction terms between sex and education showed sex differences in all cognitive scores were largest in the low education group and smallest in the high education group in China and India (*P* < 0.001 for all cognitive measures). This was also the case for orientation (*P* = 0.001) and memory (*P* = 0.02) but not fluency (*P* = 0.23) in Brazil.

**Table 3:** Comparison of sex differences in cognitive performance between education groups in each country

	Sex difference (95% CI)^a^			*P-value for interaction between sex and education* ^b^
	High education	Intermediate education	Low education	
**United States** (*N* = 13,590)				
Orientation	0.06 (−0.07, 0.19)	0.07 (−0.03, 0.17)	0.23 (0.08, 0.38)	0.18
Memory	0.21 (0.14, 0.28)	0.34 (0.28, 0.40)	0.25 (0.16, 0.35)	0.17
Attention	−0.11 (−0.17, −0.06)	−0.08 (−0.14, −0.03)	−0.17 (−0.31, −0.03)	0.29
Fluency	0.01 (−0.15, 0.16)	−0.08 (−0.18, 0.01)	−0.11 (−0.25, 0.03)	0.21
**Mexico** (*N* = 10,121)				
Orientation	−0.01 (−0.10, 0.08)	−0.02 (−0.13, 0.08)	−0.08 (−0.31, 0.16)	0.23
Memory	0.27 (0.11, 0.43)	0.27 (0.17, 0.37)	0.25 (0.06, 0.45)	0.99
Attention	−0.15 (−0.30, 0.00)	−0.28 (−0.39, −0.18)	−0.24 (−0.40, −0.08)	0.41
Fluency	0.05 (−0.14, 0.25)	−0.07 (−0.18, 0.04)	−0.02 (−0.22, 0.17)	0.98
**Brazil** (*N* = 5,432)				
Orientation	0.01 (−0.08, 0.10)	0.00 (−0.09, 0.09)	−0.21 (−0.36, −0.06)	0.001
Memory	0.17 (0.02, 0.32)	0.10 (0.01, 0.19)	−0.04 (−0.14, 0.07)	0.02
Attention	No data	No data	No data	No data
Fluency	−0.10 (−0.30, 0.10)	−0.16 (−0.25, −0.06)	−0.22 (−0.33, −0.10)	0.23
**China** (*N* = 10,226)				
Orientation	0.02 (−0.06, 0.10)	−0.08 (−0.16, −0.01)	−0.23 (−0.34, −0.12)	<0.001
Memory	0.43 (0.32, 0.54)	0.18 (0.10, 0.26)	−0.06 (−0.21, 0.08)	<0.001
Attention	0.05 (−0.04, 0.14)	−0.15 (−0.24, −0.07)	−0.14 (−0.24, −0.03)	<0.001
Fluency	No data	No data	No data	No data
**India** (*N* = 31,477)				
Orientation	−0.07 (−0.10, −0.03)	−0.20 (−0.26, −0.15)	−0.33 (−0.37, −0.29)	<0.001
Memory	0.15 (0.09, 0.22)	0.07 (0.01, 0.13)	−0.06 (−0.10, −0.02)	<0.001
Attention	−0.19 (−0.25, −0.13)	−0.38 (−0.45, −0.32)	−0.50 (−0.55, −0.46)	<0.001
Fluency	0.00 (−0.08, 0.07)	−0.09 (−0.14, −0.03)	−0.15 (−0.19, −0.11)	<0.001

^a^Estimated using weighted linear regression models adjusted for sex, marital status, age, and age^2^ and stratified by the education level.

^b^ *P*-value based on unstratified model adjusted for sex, marital status, age, age^2^, interactions between education and age terms (age, age^2^) and interaction between sex and education.

Positive values indicate females had higher cognitive scores, and negative values indicate males had higher cognitive scores.

Abbreviations: CI, confidence interval; HRS, Health and Retirement Study; MHAS, Mexican Health and Aging Study; ELSI, Brazilian Longitudinal Study of Aging; CHARLS, Chinese Health and Retirement Longitudinal Study; LASI, Longitudinal Aging Study in India.

**Figure 2:**
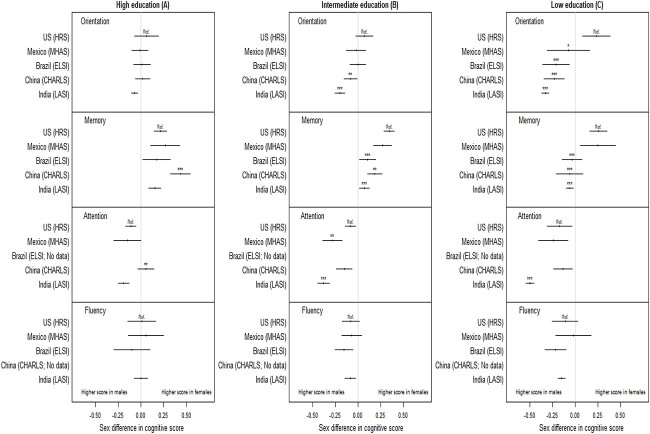
Sex differences in standardised cognitive scores in each education level and country. The left panel (**A**) shows sex differences in standardised cognitive scores in each country in the high education group. The centre panel (**B**) shows sex differences in the intermediate education group. The right panel (**C**) shows sex differences in the low education group. The confidence interval for each estimate indicates whether sex differences are statistically significant. Tests for difference with the United States (HRS) were based on pooled data; asterisks were used to indicate whether the sex difference in a given country differs from the sex difference in the United States as follows: ^*^statistically significant at α = 0.05, ^*^^*^α = 0.01, ^*^^*^^*^ α = 0.001.

Figure 2 shows results from the comparison of sex differences in cognitive function between countries within each education group. The smallest between-country differences in sex differences in cognitive function were in the high education group (Figure 2A), particularly in orientation and fluency, where sex differences were similar in all countries. Compared with the United States, sex differences in memory (*P* < 0.001) and attention (*P* = 0.003) in China were more favourable to females. In the intermediate education category (Figure 2B), sex differences were similar for fluency in all countries. Compared with the United States, there was a female disadvantage in orientation and attention in the middle-income countries and the female advantage in memory was smaller in these countries. In the low education category (Figure 2C), the between-country patterns in sex differences were similar to those in the intermediate group but the female disadvantage was more pronounced.

## Discussion

In this multicohort study of 70,846 individuals aged 60 and older from five countries, we found education, usually correlated with the economic development of a country, played an important role in sex differences in cognitive function. Compared with the United States —the high-income country in our analyses—poorer cognitive performance in females was more pronounced in middle-income countries. Females had higher scores on orientation and memory in the United States, but this was not the case in the middle-income countries where there was either no sex difference, a smaller female advantage, or a female disadvantage. Adjustment for education attenuated sex differences in all cognitive domains, highlighting its importance. The larger cognitive disadvantages in females in the middle-income countries compared with the United States were not seen in the high education group. These findings suggest that disparities in education play an important role in cognitive disadvantages observed in females compared with males, particularly in comparisons of high- and middle-income countries.

The major strength of the present study is the use of nationally representative estimates from a diverse group of countries over a narrow time period, minimising the impact of selection bias and period effect. Attention measured using serial 7s may not be appropriate for participants in settings where a considerable proportion of the study participants have no formal education. However, the use of multiple imputation allowed us to include all participants in the analysis, including those who did not perform the serial 7s task, minimising selection bias while maintaining national representativeness in all five cohort studies. In addition, we show absolute rather than relative measures of sex differences in cognitive function, allowing better interpretation of the size and comparison of sex differences between countries.

There are several limitations in this study. The cross-sectional design with data collected in a short period (2015–2019) does not allow inferences on sex differences in the rate of cognitive decline with age or birth cohort. Nonetheless there is little evidence of sex differences in cognitive decline with age in adults aged 60 and above [29], findings that were confirmed in our data which showed that sex differences in cognitive function were similar across the age span in our analyses. As such, neither age nor birth cohort effects were likely to have impacted our results. Examining differences in cognitive decline between high- and middle-income countries including consideration of birth cohort effects is an area of future research. A large proportion of participants from the middle-income countries had no formal education, precluding the use of similar education categories. Availability of more detailed education data in all cohorts, such as years of schooling, might have allowed more fine-grained analyses. We did not have data on gender to allow us to separate the effects of sex and gender. Further studies are needed to elucidate the role of gender and biological sex in a wider range of countries, ideally with nationally representative longitudinal data.

There is evidence from studies using instrumental variable approaches to address confounding that the association between education and cognitive function is causal [14, 30, 31]. Education is thought to confer a lifelong cognitive benefit because it improves cognitive function in early adulthood, thus providing a buffer against cognitive impairment at older ages [15]. As such, gender inequity in education, referring to both access to education and also gender roles that discourage women from reaching a high education level, is thought to contribute to worse cognitive function among females compared with males [8, 9, 16–20]. Our findings are consistent with this idea, as we showed that before adjustment for education, the female disadvantage in cognitive function was particularly large in countries where the education level among females was the lowest: India and China. This disadvantage was substantially attenuated when education was taken into account in the analysis. Mexico and Brazil had smaller sex differences in education and, accordingly, sex differences in cognitive function were smaller and did not substantively change after adjustment for education, possibly reflecting greater investment in education in these two countries [19, 32, 33]. Sex differences in cognitive function unaccounted for by education are likely attributable to a combination of sex differences in biological and other social, lifestyle, and health factors, such as chronic disease, income, and occupation [34].

Our results agree with previous studies showing sex differences in orientation vary by country [9], a consistent female advantage in memory in high-income countries [10] that is reversed in middle-income countries [20], and a female disadvantage on tasks of sustained attention [7] including serial 7s [9]. Previous studies undertaken in high-income countries also indicated no sex differences in fluency after taking education into account [7], or a female disadvantage only in the low education group [17]. Consistent with these studies, we found that female disadvantage in fluency was eliminated in all countries in the high education group. We build on the existing evidence by showing that attenuation of female disadvantage in cognitive function among those who were most educated occurred across cognitive domains and in middle- and high-income countries. Sex differences tended to be largest and least favourable to females in the low education group, with the biggest difference between middle-income countries and the United States. In contrast, in the high education group there were either no or few sex differences. In this group, sex differences in the middle-income countries were mostly similar to those in the United States. These findings suggest that the larger female disadvantage in cognitive function in the middle-income countries compared with the United States is likely to be mainly driven by those in the low education group.

These findings suggest that improving education could contribute to reducing the female disadvantage in cognitive function. In the middle-income countries, 8–9 years of schooling was sufficient to see the same pattern of sex differences as in the high education category in the United States, which was composed of participants with college/university education. Being highly educated compared with the rest of the population may open up otherwise inaccessible occupational and lifestyle pathways that further contribute to cognitive health [35], compounding the effect of education on cognitive function in old age. Furthermore, sex inequalities in education have decreased in many middle- and high-income countries over the 20th century [36]. These changes may eventually result in smaller sex inequalities in cognitive function in middle- and high-income countries.

This study reiterates the role of education in cognitive function and further shows that larger sex disparities in education in middle-income countries may account for the larger female cognitive disadvantage seen in middle- compared with high-income countries. Despite growing evidence that education plays an important role in cognitive function, current guidelines for cognitive ageing and dementia do not include education as a target to address inequities [37, 38]. Access to education for all, including gender parity in education, is a United Nations sustainable development goal [39]. Considering the beneficial effect of education on cognitive function and dementia risk, national and international policies aiming to improve cognitive health should prioritise the promotion of more education for all, with an emphasis on gender equity.

## Supplementary Material

aa-22-1185-File002_afad019
