# Supplementary material for: Comparison of sex differences in cognitive function in older adults between high- and middle-income countries and the role of education: a population-based multicohort study
Source: Age Ageing. 2023 Feb 21;52(2):afad019. doi: 10.1093/ageing/afad019 (PMC9949595; doi:10.1093/ageing/afad019)
Supplement: aa-22-1185-File002_afad019 [file aa-22-1185-file002_afad019.pdf]

<u>Comparison of sex differences in cognitive function in older adults between high- and middle-income countries and the role of education: a population-based multicohort study</u>

SUPPLEMENTARY MATERIALS

Appendix 1. Education categories in the five countries. 2
Appendix 2. Description of included cognitive tests. 3
Appendix 3. Procedure used for multiple imputation 4
Appendix 4. The number and percentage of imputed data on covariates and cognitive scores in each cohort. 5
Appendix 5. Characteristics of men and women in the five countries: observed data. 6
References 7

Appendix 1. Education categories in the five countries.

Education category	Country (cohort)									
	US (HRS)		Mexico (MHAS)		Brazil (ELSI)		China (CHARLS)		India (LASI)	
	Approximate years of schooling	%[a]	Approximate years of schooling	%[a]	Approximate years of schooling	%[a]	Approximate years of schooling	%[a]	Approximate years of schooling	%[a]
Low	<12	13.4	<6	22.0	<2	27.6	<6	30.1	0	60.3
Intermediate	12	50.8	6-11	51.2	2-8	51.7	6-8	42.1	1-7	20.3
High	>12	35.8	>11	26.8	>8	20.7	>8	27.8	>7	19.4

[a]Data are percentages in the weighted, imputed dataset.

Abbreviations: HRS, Health and Retirement Study; MHAS, Mexican Health and Aging Study; ELSI, Brazilian Longitudinal Study of Aging; CHARLS, Chinese Health and Retirement Longitudinal Study; LASI, Longitudinal Aging Study in India

Appendix 2. Description of included cognitive tests.

Four cognitive domains were examined: *orientation,* assessed using the date naming task, requiring participants to correctly name the day, month, and year that the interview took place; *episodic memory,* using immediate and delayed recall, requiring participants to recall either an 8-word (MHAS) or 10-word (all other cohorts) list, once immediately after hearing the list and then again after some time; *attention,* using the serial 7s test where participants were asked to count backward from 100 by 7 five times; and *verbal fluency* using the animal naming task requiring participants to list as many animal names as possible within a minute. Orientation and episodic memory were available in all cohorts. Attention was not tested in ELSI and verbal fluency was not tested in CHARLS.

Appendix 3. Procedure used for multiple imputation

The aim of the imputation was to include all participants in the analyses so that survey weights could be applied to yield nationally representative estimates. Missing data in all variables were imputed using predictive mean matching in multiple imputation with chained equations. The number of imputations was determined using the 'linear rule of thumb' which is appropriate when up to 50% of data are missing.[1 2] The decision on the number of imputations was based on 100 minus the percentage of complete cases, plus an additional 10 imputations to be conservative, using the cohort with the greatest proportion of missing data. In our case, this led to 50 imputations in each cohort.

The imputation was undertaken separately in men and women in each cohort to allow us to include sex and cohort interaction terms in the main analysis. The variables used in the imputation were: 1) the same as in the main analysis: all cognitive tests, age, age^2, marital status, education, and interactions of education with age and age^2; 2) auxiliary variables (not used in the main analysis but informative for the missingness pattern): number of limitations in basic/instrumental activities of daily living and mobility activities, and labour force status (employed, unemployed, retired, homemaker); and 3) survey weights, included using interaction terms between survey weights and each variable in the imputation model in order to correctly specify the imputation model.

Appendix 4. The number and percentage of imputed data on covariates and cognitive scores in each cohort.

Cohort (Total N, % complete cases)	The number (%) of imputed data							
	Covariates			Cognitive scores				
	Age	*Marital status*	*Education*	*Orientation*	*Immediate recall*	*Delayed recall*	*Serial 7s*	*Verbal fluency*
HRS (13590, 61.0)	0 (0.0)	0 (0.0)	4 (0.0)	4372 (32.1)	905 (6.7)	906 (6.7)	1607 (11.8)	4659 (34.3)
MHAS (10121, 81.9)	1 (0.0)	0 (0.0)	468 (4.6)	1019 (10.0)	1172 (11.6)	1172 (11.6)	1154 (11.4)	1323 (13.1)
ELSI (5432, 95.7)	0 (0.0)	0 (0.0)	51 (0.9)	290 (5.3)	290 (5.3)	290 (5.3)	No data	303 (5.6)
CHARLS (10226, 58.4)	46 (0.5)	0 (0.0)	0 (0.0)	1093 (10.7)	2189 (21.4)	2619 (25.6)	3665 (35.8)	No data
LASI (31477, 57.7)	0 (0.0)	0 (0.0)	0 (0.0)	747 (2.4)	1122 (3.6)	3528 (11.2)	12022 (38.2)	887 (2.8)

Abbreviations: HRS, Health and Retirement Study; MHAS, Mexican Health and Aging Study; ELSI, Brazilian Longitudinal Study of Aging; CHARLS, Chinese Health and Retirement Longitudinal Study; LASI, Longitudinal Aging Study in India

Appendix 5. Characteristics of men and women in the five countries: observed data.

	US (HRS) N = 13590			Mexico (MHAS) N = 10121			Brazil (ELSI) N = 5432			China (CHARLS) N = 10226			India (LASI) N = 31477		
	Men	Women	P-value	Men	Women	P-value	Men	Women	P-value	Men	Women	P-value	Men	Women	P-value
	41.6%	58.4%		44.1%	55.9%		40.0%	60.0%		48.7%	51.3%		48.0%	52.0%	
Age, Mean	72.2	73.0	<0.001	72.0	71.6	0.01	70.1	70.5	0.05	69.1	69.3	0.28	69.0	68.7	<0.001
Age group															
60-69	45.7	43.4		41.7	45.6		54.4	52.0		59.9	59.6		59.3	61.2	
70-79	31.1	30.9	0.002	40.1	36.2	<0.001	31.9	33.4	0.22	30.2	29.2	0.23	30.1	27.8	<0.0001
80+	23.2	25.7		18.2	18.2		13.8	14.6		9.7	10.6		10.5	11.0	
Married/cohabiting															
Yes	75.0	46.8	<0.001	78.0	48.5	<0.001	71.8	37.7	<0.001	86.2	70.5	<0.001	82.8	46.4	<0.001
No	25.0	53.2		22.0	51.5		28.2	62.3		13.8	29.5		17.2	53.6	
Education															
Low	18.4	19.0		15.8	20.3		30.2	31.1		15.1	49.6		36.3	69.7	
Intermediate	48.6	54.7	<0.001	50.9	52.0	<0.001	50.1	49.7	0.77	50.9	35.7	<0.001	29.7	18.8	<0.001
High	32.9	26.3		28.0	23.6		18.7	18.3		34.0	14.6		34.1	11.5	

Data shown are percentages unless otherwise indicated.

Abbreviations: HRS, Health and Retirement Study; MHAS, Mexican Health and Aging Study; ELSI, Brazilian Longitudinal Study of Aging; CHARLS, Chinese Health and Retirement Longitudinal Study; LASI, Longitudinal Aging Study in India

References

1. Bodner TE. What Improves with Increased Missing Data Imputations? *STRUCT EQU MODELING* 2008;15(4):651-75. doi: 10.1080/10705510802339072
2. van Buuren S. Flexible imputation of missing data / by Stef van Buuren. Second edition. ed: Boca Raton, FL : Chapman and Hall/CRC, an imprint of Taylor and Francis 2018.
